# A New Treatment for Fistulæ

**Published:** 1900-06

**Authors:** W. T. Campbell

**Affiliations:** Cincinnati, O.


### REPORTS OF CASES.

### A NEW TREATMENT FOR FISTULÆ.

By W. T. Campbell, V.S.,
CINCINNATI, O.

It has been a long-felt want among veterinarians to find some remedy which will be useful in cases of fistula that would be of a less irritating nature than our caustics and still have a stronger action than our mild antiseptics. Being in a region where fistula is very common, I came in contact with a great many cases, and as a result have tried every treatment that I could possibly think of, and cannot say that I have met with great success.

Having by a mere accident come in possession of protargol, I gave it a trial, and found it to be just the thing for such cases. It is a yellowish light powder, soluble in water, and is a derivative of silver. Although not as irritating as the nitrate, it still has the same action as a bactericide and antiseptic.

As it may be interesting to some of the readers, I will describe a few cases in which I used it and show how it acts.

On January 24th I was called to see a roan mare, four years old, with a very bad case of fistulous withers, having been caused by a heavy team saddle, and had been standing about two months. The withers were swollen on each side to the size of an ordinary ham, with an ugly running sore on the top, from which came a very fetid discharge.

After making an opening at the bottom I inserted a seton and a few plugs of bichloride of mercury and nitrate of silver, which gave me an abundant discharge of pus in about ten hours. About this time I came to the conclusion I would use my protargol, and on the following day I started to syringe the wound with a solution as follows: Protargol, 10 parts; glycerin, 50 parts; water, 40 parts. This was injected three times a day, and hot woollen rags were laid over the withers every hour. On February 1st the swelling had gone down considerable, and the discharge was less fetid. On the 10th she was able to drive, and on the 15th was entirely well, and as yet there has been no recurrence.

A gray horse, belonging to a coal firm, came to the hospital with fistula which seemed to be one mass of pipes. I made an opening at the bottom, and into this I injected the above solution, and was surprised at the amount of pus it brought out. I continued this three times a day for three weeks, and its effects were wonderful, as it dried up the fistula and healed the wound in just that time.

Although not generally classed as fistula, I consider a quittor just the same, and, meeting with good success in a late case, I shall add it to the list.

On February 1st I was called to see a horse which had suffered from a moist corn, and as a result of this had opened at the top of the foot, from which the flow of pus was constant. I removed the shoe and pared out the corn, giving it a free opening, after which I ran a seton from the bottom to the top of the foot. I then gave it a covering of the powdered protargol, and injected the solution in from the bottom three times a day. Under this treatment he progressed nicely, and in three weeks was entirely well. In all these cases the regular course of internal medicines was administered, and all I claim of interest is the action of this solution on fistulous tracts, and to those who have such cases it would be well to try it, as in every case in which I have used it my success was greater and cure more permanent and in less time than any other treatment I have ever used.

